# N-Terminus of GRXCR2 Interacts With CLIC5 and Is Essential for Auditory Perception

**DOI:** 10.3389/fcell.2021.671364

**Published:** 2021-05-05

**Authors:** Jinan Li, Chang Liu, Bo Zhao

**Affiliations:** Department of Otolaryngology-Head and Neck Surgery, Indiana University School of Medicine, Indianapolis, IN, United States

**Keywords:** hearing loss, hair cell, stereocilia, CLIC5, GRXCR2

## Abstract

Stereocilia of cochlear hair cells are specialized mechanosensing organelles that convert sound-induced vibration to electrical signals. Glutaredoxin domain-containing cysteine-rich protein 2 (GRXCR2) is localized at the base of stereocilia and is necessary for stereocilia morphogenesis and auditory perception. However, the detailed functions of GRXCR2 in hair cells are still largely unknown. Here, we report that GRXCR2 interacts with chloride intracellular channel protein 5 (CLIC5) which is also localized at the base of stereocilia and required for normal hearing in human and mouse. Immunolocalization analyses suggest that GRXCR2 is not required for the localization of CLIC5 to the stereociliary base during development, or vice versa. Using clustered regularly interspaced short palindromic repeats (CRISPR)/Cas9 system, we deleted 60 amino acids near the N-terminus of GRXCR2 essential for its interaction with CLIC5. Interestingly, mice harboring this in-frame deletion in *Grxcr2* exhibit moderate hearing loss at lower frequencies and severe hearing loss at higher frequencies although the morphogenesis of stereocilia is minimally affected. Thus, our findings reveal that the interaction between GRXCR2 and CLIC5 is crucial for normal hearing.

## Introduction

Hearing loss is the fourth leading cause of disability in the world, affecting 6–8% of the population (Brown et al., 2018). The most common form is sensorineural hearing loss, frequently caused by morphogenetic defects of cochlear hair cells, the sensors in the mammalian inner ear. Hair cells convert mechanical sound stimuli into electrical signals transmitted to the nervous system (Gillespie and Muller, 2009; Pacentine et al., 2020). Mutations that cause deafness frequently are linked to defects of the stereociliary hair bundle, the staircase-shaped cellular organelles that protrude from the apical surface of hair cells (Frolenkov et al., 2004).

**FIGURE 1:**
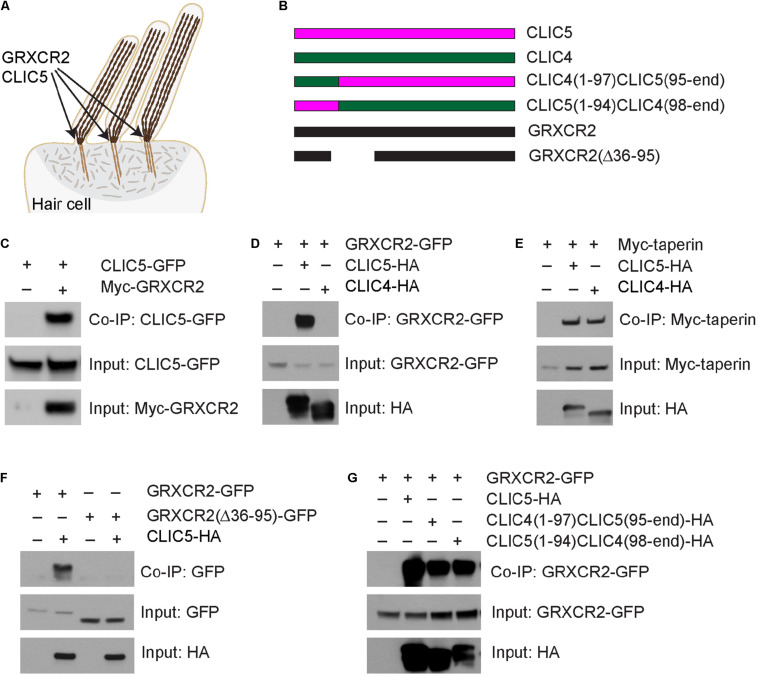
Glutaredoxin domain-containing cysteine-rich protein 2 (GRXCR2) interacts with CLIC5. **(A)** Diagram of cochlear hair cell showing the staircase-shaped hair bundle. Inside the stereocilia, the tightly cross-linked actin filaments provide the stiffness of stereocilia. GRXCR2 and CLIC5 are concentrated at the base of stereocilia, the site of stereocilia pivoting. **(B)** Diagram of the constructs used for biochemical experiments. **(C–G)** HEK293 cells were transfected with the constructs indicated on the top of each panel. Immunoprecipitations were carried out with Myc-antibody **(C)** or HA-antibody **(D–G)** followed by western blotting to detect co-expressed proteins. The upper rows show Co-IP results and lower rows show input proteins.

The base of stereocilia, the site of stereocilia pivoting, shows a striking structural organization, including the formation of a taper, as well as cytoskeletal specializations, including rootlet filaments (Figure 1A; Frolenkov et al., 2004; Pacentine et al., 2020). Genetic and immunolocalization studies have identified several proteins linked to hearing loss in humans that are concentrated at or near the basal region of stereocilia (Pacentine et al., 2020). TRIOBP bundles actin filament and forms rootlet which provides the durability and rigidity for mechanosensitive of stereocilia (Kitajiri et al., 2010; Katsuno et al., 2019). VLGR1 interacts with USH2A, whirlin and PDZD7 and makes up the ankle links that connect the stereocilia at their base during development (Michalski et al., 2007; Grati et al., 2012; Chen et al., 2014; Morgan et al., 2016). Taperin, an actin regulatory protein (Rehman et al., 2010; Liu et al., 2018), forms a dense-core-like structure which is encircled by a circumferential ring structure formed by RIPOR2 (previously named as Fam65b) oligomers at the taper region of stereocilia (Zhao et al., 2016). Chloride intracellular channel protein 5 (CLIC5), a ∼28 kDa highly conserved protein in vertebrates, associates with taperin, radixin and myosin 6 and stabilizes membrane-actin filament linkages (Salles et al., 2014). Although mutations of CLIC5 leads to hearing loss in humans and mice (Gagnon et al., 2006; Seco et al., 2015), the underlying molecular mechanism is unknown.

Glutaredoxin domain-containing cysteine-rich protein 2 (GRXCR2), mutations of which lead to hearing loss in humans and mice (Imtiaz et al., 2014; Avenarius et al., 2018; Liu et al., 2018; Wonkam et al., 2021), also localizes at the base of stereocilia (Liu et al., 2018). Loss of GRXCR2 leads to the mislocalization of taperin and disorganization of stereocilia, which result in profound hearing loss in mice. Remarkably, reducing taperin expression level by depleting one allele of taperin in the genome rescues the morphological defects of stereocilia but only partially restores the hearing of null *Grxcr2*-mutant mice, suggesting that GRXCR2 interacts with additional protein(s) which are also required for normal hearing (Liu et al., 2018).

In this study, we found that GRXCR2 interacts with CLIC5. The localization of these two proteins at the stereociliary base is independent of each other. Disrupting the interaction between GRXCR2 and CLIC5 *in vivo* has minimal effects on stereocilia morphogenesis but leads to hearing loss in mice. Thus, the interaction between GRXCR2 and CLIC5 is crucial for normal hearing.

## Materials and Methods

### Yeast Two-Hybrid Screening

Cochlear specific yeast two-hybrid cDNA library constructed from mRNA isolated from P3-P7 mouse organ of Corti was constructed previously (Zhao et al., 2014) and yeast two-hybrid screening was performed following the manufacturer’s instruction (Dualsystems Biotech). In brief, *Clic5* cDNA (NM_172621) was cloned into a yeast two-hybrid bait vector and introduced into yeast by transformation. Self-activation of the bait was tested and screening stringency was optimized. Yeast libraries were then transformed into yeast expressing the bait. Putative interactors were selected from among ∼1 million transformants. Positive clones were picked and re-tested for bait dependency. Plasmids from positive clones were isolated and the identity of interacting partners was determined by DNA sequencing.

### Cell Culture

HEK293 cell line was obtained from ATCC. Cells were maintained in the DMEM medium (Cat# 11965118, Thermo Fisher Scientific) supplemented with 10% heat-inactivated fetal bovine serum (Fisher Scientific), 100 U/ml penicillin and 100 μg/ml streptomycin (Fisher Scientific). Cells were grown at 37°C in a 5% CO_2_ humidified atmosphere.

### cDNA Constructions, Immunoprecipitations and Western Blots

The coding sequence of *Clic5*, *Clic4*, *Grxcr2*, and *taperin* were amplified from mouse cochlear cDNA library. Expression of the constructs, immunoprecipitations, and western blots were carried out as described (Senften et al., 2006; Zhao et al., 2016; Liu et al., 2018). Immunoprecipitation experiments were carried out at least three times to verify the reproducibility of the data. The following antibodies were used for the experiments: anti-HA (Cat# 2367S, Cell signaling); anti-Myc (Cat# 2278S, Cell signaling); anti-Myc (Cat# sc-40, Santa Cruz); anti-GFP (Cat# sc-9996, Santa Cruz).

### Animal Models and Animal Care

*Grxcr2*^–/–^ mouse (previously named as *Grxcr2^*D*46/D46^* mouse) has been described previously (Liu et al., 2018). *Clic5*^–/–^ mouse (also named as *Clic5*^*jbg/jbg*^ mouse) was purchased from Jackson lab (Gagnon et al., 2006). Using clustered regularly interspaced short palindromic repeats (CRISPR)/Cas9 technology, 180 bp nucleotides in exon 1 of *Grxcr2* were deleted from the genome of C57BL/6J mouse, resulting in a change of amino acid sequence after residue 35, a loss of 60 amino acids and remains in frame. In brief, two sgRNAs (5′-GGATGGCGTTTATGGGTCTGGGG-3′ and 5′-CAGCGGCGCCTACACTCTGGCGG-3′) were synthesized by *in vitro* transcription and microinjected into one-cell embryos. Genomic DNA was then collected from the offspring obtained by the embryo injections, screened using PCR and then sequenced to confirm in-frame deletion. The founder mice were then back-crossed with C57BL/6J mice for two generations. To genotype the *Grxcr2^*D*180/D180^* mice, the following primers were used: 5′-TCTTCCTACAGTGGCCGAGT-3′ and 5′-TGAATGTGAGCGAGATACCG-3′. All animal experiments were approved by Institutional Animal Care and Use Committee of The Scripps Research Institute and Indiana University School of Medicine. Both male and female mice were used in our experiment, and we did not find any sex-based differences.

### Whole Mount Immunostaining

Cochlear whole mount staining was carried out as described (Zhao et al., 2016; Liu et al., 2018). In brief, organ of Corti tissue was dissected and fixed in 4% PFA in Hank’s Balanced Salt Solution (HBSS) for 20 min. Samples were blocked for 20 min at room temperature in HBSS containing 5% bovine serum albumin (BSA), 1% goat serum and 0.5% Triton X-100, and then incubated overnight at 4°C with primary antibodies in HBSS containing 1% BSA and 0.1% Triton X-100. Samples were washed in HBSS and incubated 2 h at room temperature with secondary antibodies. Tissues were mounted in ProLong^®^ Antifade Reagents (Invitrogen). Stacked images (Z step, ∼0.17 μm; pixel size, 0.04 μm) were then captured by deconvolution microscope (Leica) using a 100 X objective (HCX PL APO 100×/1.40–0.70 OIL). Images were then deconvoluted using blind deconvolution method. Primary antibodies were as follows: anti-GRXCR2 (Cat# HPA059421, Sigma); anti-taperin (Cat# HPA020899, Sigma); anti-CLIC5 (Cat# ACL-025, Alomone labs). Additional reagents were: Alexa Fluor 488-phalloidin (Thermo Fisher Scientific), Alexa Fluor 568-phalloidin (Thermo Fisher Scientific), Alexa Fluor 647-phalloidin (Thermo Fisher Scientific), Alexa Fluor 488 goat anti-rabbit (Thermo Fisher Scientific), and Alexa Fluor 546 goat anti-rabbit (Thermo Fisher Scientific).

### Scanning Electron Microscopy

The experiments were performed as described (Zhao et al., 2016; Liu et al., 2018). In brief, inner ears were dissected in fixative (2.5% glutaraldehyde; 4% formaldehyde; 0.05 mM Hepes Buffer pH 7.2; 10 mM CaCl_2_; 5 mM MgCl_2_; 0.9% NaCl) and fixed for 1 h at RT. Samples were then dissected to remove the stria vascularis, Reissner’s membrane and tectorial membrane. Samples were post-fixed by immersion in for 1 day in the same fixative at 4°C. After fixation in 1% OsO_4_ for 1 h, samples were serially dehydrated in ethanol, dried in a critical point drier (Autosamdri-815A, Tousimis), finely dissected and mounted on aluminum stubs. Samples were then coated by gold and viewed on a JEOL 7800F scanning electron microscope. At least three animals representative of each experimental paradigm were analyzed.

### Auditory Brainstem Response Measurement

Auditory brainstem responses (ABRs) of mice were recorded as described (Zhao et al., 2016; Liu et al., 2018) using TDT Bioacoustic system 3 and BioSigRZ software. In brief, mice were anesthetized using the mixture of 100 mg/kg ketamine and 10 mg/kg xylazine. Electrodes were inserted under the skin at the vertex and ipsilateral ear, while a ground was inserted under the skin near the tail. The speaker was placed 5 cm away from the mouse ear. Tone stimulus is presented 21 times per second. Band-pass filtered from 300 to 3000 Hz. Averaging window was 10 ms. A total of 512 responses were averaged at each frequency and level combination. The intensity of sound stimulus was started at 90 dB SPL and decreased in 10 dB SPL stepwise to a sub-threshold level. ABR thresholds were analyzed for both ears and for a range of frequencies (for Pure Tone, 4–28 kHz). If no ABR wave was detected at maximum intensity stimulation, a nominal threshold of 90 dB was assigned.

### Quantification and Statistical Analysis

All data are mean ± standard error of the mean. Student’s two-tailed unpaired *t* test or Two-way ANOVA were used to determine statistical significance (^∗^, *p* < 0.05, ^∗∗^, *p* < 0.01, ^∗∗∗^, *p* < 0.001).

## Results

### GRXCR2 Interacts With CLIC5

Chloride intracellular channel protein 5 has been linked to sensorineural hearing loss in humans and mice (Gagnon et al., 2006; Salles et al., 2014; Seco et al., 2015). However, the molecular functions of CLIC5 in hair cells are still unknown. To identify binding partners for CLIC5, an unbiased yeast-two-hybrid screening was performed using full length CLIC5 as bait. By screening a yeast-two-hybrid library constructed using RNA extracted from organ of Corti, we identified 30 positive clones from ∼1 million transformants. One positive clone expresses taperin, consistent with the previous finding that CLIC5 interacts with taperin (Salles et al., 2014; Bird et al., 2017). Interestingly, 23 positive clones express GRXCR2, suggesting a strong interaction between CLIC5 and GRXCR2. To confirm the yeast-two-hybrid data, we carried out co-immunoprecipitation (co-IP) experiments with extracts from HEK293 cells that were transfected with GFP-tagged CLIC5 and Myc-tagged GRXCR2. CLIC5-GFP was co-immunoprecipitated with Myc-GRXCR2 (Figure 1C). Correspondingly, GFP tagged GRXCR2 was co-immunoprecipitated with HA-tagged CLIC5 (Figure 1D).

Chloride intracellular channel protein 5 shows an overall 75% similarity to its paralog CLIC4. CLIC5 and CLIC4 are both highly expressed in the cochlear hair cells and concentrated at the base of stereocilia (Shen et al., 2015; Bird et al., 2016). Although both of them interact with taperin (Figure 1E and Bird et al., 2017), only CLIC5 is known to be essential for the morphogenesis of stereocilia and auditory perception (Bird et al., 2016), suggesting that in addition to TPRN, CLIC5, and CLIC4 have different binding partners and functions. Interestingly, our data shows that only CLIC5 binds strongly to GRXCR2 (Figure 1D), suggesting that the specific interaction between CLIC5 and GRXCR2 might be important for the auditory perception. To identify region(s) in GRXCR2 critical for the interaction with CLIC5, we next generated several truncated GRXCR2 constructs and their interactions with CLIC5 were evaluated by co-IP (Supplementary Figure 1A). Finally, we found that 60 amino acids from 36 to 95 in GRXCR2, which are highly conserved in mammals, are essential for its interaction with CLIC5 (Figure 1F). A previous study reported that amino acids from 121 to 140 in GRXCR2 mediate the interaction with taperin (Liu et al., 2018). Deleting 60 amino acids from 36 to 95 in GRXCR2 did not affect its binding with taperin (Supplementary Figure 1B), further confirming that CLIC5 and taperin bind to different regions of GRXCR2. To identify region(s) in CLIC5 mediating the interaction with GRXCR2, we generated domain-swapped CLIC5 and CLIC4 proteins by exchanging their N terminal 94 aa motifs (Figure 1B and Supplementary Figure 1C). Interesting, both chimeric proteins interacted with GRXCR2 (Figure 1G), suggesting that at least two regions in CLIC5 are essential for its interaction with GRXCR2.

### Independent Localization of CLIC5 and GRXCR2 in Hair Cells During Development

**FIGURE 2:**
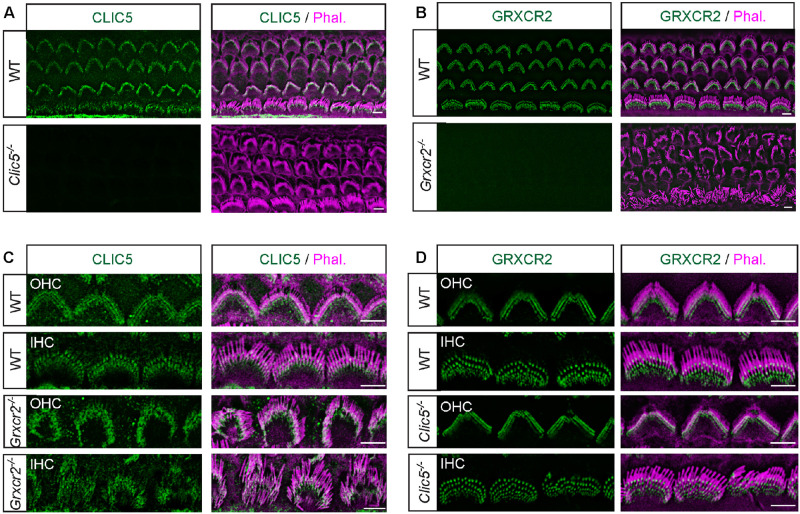
Independent localization of CLIC5 and GRXCR2 in hair cells during development. **(A)** Costaining of cochlear whole mounts from wild-type and *Clic5*^–/–^ mice at postnatal day 4 (P4) with CLIC5-antibody (green) and phalloidin (red) to reveal stereocilia. Note, no immunostaining signal in *Clic5*^–/–^ hair cells, suggesting that the CLIC5-antibody is specific. **(B)** Costaining of cochlear whole mounts from wild-type and *Grxcr2*^–/–^ mice with GRXCR2-antibody and phalloidin. Note, no immunostaining signal in *Grxcr2*^–/–^ hair cells, suggesting that the GRXCR2-antibody is specific. **(C)** Co-staining of P4 cochlear whole mounts from wild-type and *Grxcr2*^–/–^ mice with CLIC5-antibody and phalloidin. Note, CLIC5 was concentrated at the base of stereocilia in outer hair cells (OHCs) and inner hair cell (IHCs) of wild-type and *Grxcr2*^–/–^ mice. **(D)** Co-staining of P5 cochlear whole mounts from wild-type and *Clic5*^–/–^ mice with GRXCR2-antibody and phalloidin. Note, GRXCR2 was concentrated at the base of stereocilia in wild-type and *Clic5*^–/–^ hair cells. Scale bars: 5 μm.

Previous studies found that GRXCR2 is essential for the localization of taperin to the basal region of stereocilia (Liu et al., 2018). CLIC5 is also localized at the base of stereocilia and forms a complex with taperin (Gagnon et al., 2006; Salles et al., 2014). To investigate the extent to which the interaction between GRXCR2 and CLIC5 is required for their proper localization in the stereocilia, immunohistochemistry experiments were performed. To confirm the immunostaining signals obtained are specific, antibodies used in this study were validated using samples from null mutant mice. In the wild-type hair cells, we observed expression in outer hair cells (OHCs) and inner hair cells (IHCs) where CLIC5 and GRXCR2 immunostaining signals concentrated at the base of each stereocilium, outlining the shape of stereociliary bundle (Figures 2A,B). In the hair cells from *Clic5*^*jbg/jbg*^ mouse (referred as *Clic5*^–/–^ mouse hereafter), in which a 97 bp deletion introduces a translational frameshift and premature stop codon in *Clic5* (Gagnon et al., 2006), no immunostaining signal of CLIC5 was observed, suggesting that the immunostaining signals obtained using CLIC5 antibody are specific (Figure 2A). Similarly, the GRXCR2 antibody used in this study is also specific as no immunostaining signal was observed in the hair cells from *Grxcr2^*D*46/D46^* mouse (Liu et al., 2018, referred as *Grxcr2*^–/–^ mouse hereafter), in which a 46 bp frameshift deletion creates a premature stop codon in *Grxcr2* (Figure 2B).

At higher magnifications, CLIC5 localized at the base of stereocilia in wild-type OHCs and IHCs, consistent with previous results (Gagnon et al., 2006; Salles et al., 2014). In *Grxcr2*^–/–^ hair cells, CLIC5 was still concentrated at the base of stereocilia, suggesting that GRXCR2 is not essential for the localization of CLIC5 to the basal region of stereocilia during development (Figure 2C). Our previous study found that taperin is diffused along the stereocilia length and sometimes accumulated toward the distal end of the stereocilia in the *Grxcr2*^–/–^ hair cells (Liu et al., 2018). Although taperin interacts with CLIC5 at the base of stereocilia (Salles et al., 2014; Bird et al., 2016, 2017), mislocalized taperin in the *Grxcr2*^–/–^ hair cells did not affect the localization of CLIC5 in stereocilia. Similarly, GRXCR2 localized at the base of stereocilia in both wild-type and *Clic5*^–/–^ hair cells, suggesting that CLIC5 is not required for the GRXCR2 localization in hair cells during development either (Figure 2D).

### Disrupting the Interaction Between GRXCR2 and CLIC5 Has Minimal Effects on Stereocilia Morphogenesis

**FIGURE 3:**
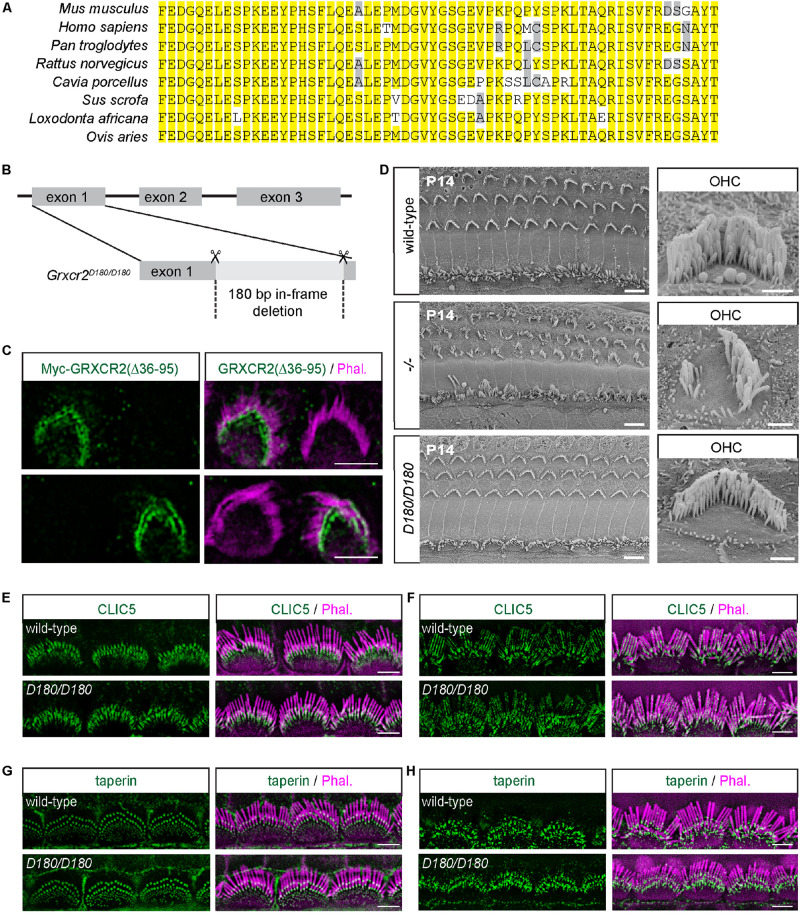
Disrupting the interaction between GRXCR2 and CLIC5 has minimal effects on stereocilia morphogenesis. **(A)** Amino acids from 36 to 95 in GRXCR2 are highly conserved in mammals. **(B)** Diagram of the strategy to generate *Grxcr2^*D*180/D180^* mice. sgRNAs targeting exon 1 of *Grxcr2* induced a 180-bp in-frame deletion, which was confirmed by sanger sequencing. **(C)** P3 cochlear explants were injectoporated to express Myc-GRXCR2 (Δ36–95). Two days later, tissues were fixed and stained with the Myc-antibody. Note Myc-GRXCR2 (Δ36–95) was concentrated at the base of the stereocilia. **(D)** Scanning electron microscope images showing auditory sensory epithelia of wild-type (upper panel), *Grxcr2*^–/–^ (middle panel), and *Grxcr2^*D*180/D180^* (lower panel) mice at the age of P14. Note, stereocilia of *Grxcr2*^–/–^ mice were disorganized while the stereocilia were fairly normal in *Grxcr2^*D*180/D180^* mice. Scale bars: left panel 5 μm, right panel 1 μm. **(E,F)** Cochlear whole mounts from wild-type and *Grxcr2^*D*180/D180^* mice at the age of P7 **(E)** and 6 weeks **(F)** were stained for CLIC5. Stereocilia were visualized by staining with phalloidin (red). Note, CLIC5 staining did not show any significant change in *Grxcr2^*D*180/D180^* hair cells. **(G,H)** Cochlear whole mounts from wild-type and *Grxcr2^*D*180/D180^* mice at the age of P7 **(G)** and 6 weeks **(H)** were stained for taperin. Note, taperin was still concentrated at the base of stereocilia in *Grxcr2^*D*180/D180^* hair cells. Scale bars: 5 μm.

The 60 amino acids from 36 to 95 in GRXCR2, the binding site with CLIC5, are highly conserved in mammals (Figure 3A). To investigate the extent to which the interaction between GRXCR2 and CLIC5 is involved in auditory perception, 180 bp nucleotides coding these 60 amino acids were deleted from the exon 1 of *Grxcr2* using the CRISPR/Cas9 system. Founder mouse was back-crossed with wild-type C57BL/6J mice for two generations to reduce potential off-target effects of CRISPR. Finally, we obtained a new *Grxcr2*-mutant mouse line, which will be referred as *Grxcr2^*D*180/D180^* mouse (Figure 3B). To confirm whether the 180 bp is deleted from the mRNA of *Grxcr2*, mRNA was extracted from the inner ear of *Grxcr2^*D*180/D180^* mice. Then cDNA of *Grxcr2* was amplified and sequenced. Indeed, the 180 bp nucleotides were deleted from *Grxcr2*. The GRXCR2 antibody used for immunostaining does not recognize GRXCR2 (Δ36–95) as the immunogen of the antibody is located within the first 80 amino acids of GRXCR2. To investigate whether GRXCR2 (Δ36–95) is also concentrated at the base of stereocilia, injectoporation (Xiong et al., 2014) was performed to express Myc-tagged GRXCR2 (Δ36–95) in P3 hair cells. Two days after injectoporation, hair cells were fixed and Myc-antibody was used to detect Myc-GRXCR2 (Δ36–95). Similar to the full-length GRXCR2, GRXCR2 (Δ36–95) was also concentrated near the base of the stereocilia (Figure 3C).

To analyze the stereocilia morphology in *Grxcr2^*D*180/D180^* mice, scanning electron microscopy (SEM) was performed. In *Grxcr2*^–/–^ mice, most of the hair cell bundles were disorganized and had lost their characteristic V-shapes, which is consistent with the previous results (Figure 3D; Liu et al., 2018). Interestingly, in *Grxcr2^*D*180/D180^* mice, both IHC and OHC bundles were minimally affected at P14, suggesting that the interaction between GRXCR2 and CLIC5 is not essential for the stereocilia morphogenesis (Figure 3D).

To investigate the localization of CLIC5 in the *Grxcr2^*D*180/D180^* hair cells, whole mount immunostaining was performed. Similar to that in wild-type and *Grxcr2*^–/–^ hair cells, CLIC5 was also concentrated at the base of stereocilia in *Grxcr2^*D*180/D180^* hair cells at P7 (Figure 3E). In the 6-week-old wild-type hair cells, some CLIC5 entered stereocilia shaft. The immunostaining signals of CLIC5 had no significant difference between wild-type and *Grxcr2^*D*180/D180^* hair cells (Figure 3F). These results further suggest that GRXCR2 is not required for the localization of CLIC5 to the stereociliary base. Deleting 60 amino acids from 36 to 95 in GRXCR2 does not affect the binding of GRXCR2 with taperin. Consistently, in the *Grxcr2^*D*180/D180^* hair cells, taperin was still concentrated at the base of stereocilia in both P7 and adult hair cells (Figures 3G,H).

### Disrupting the Interaction Between GRXCR2 and CLIC5 Leads to Hearing Loss in Mice

**FIGURE 4:**
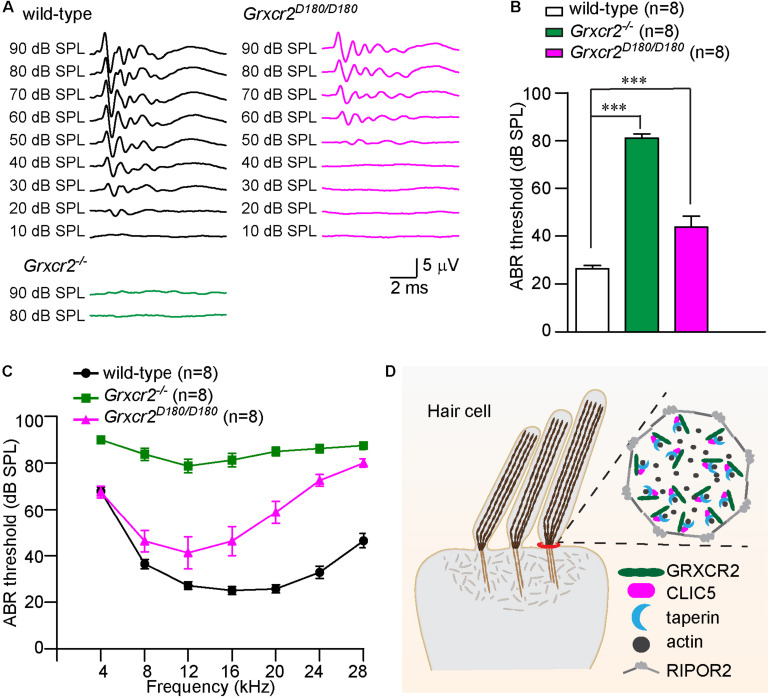
Disrupting the interaction between GRXCR2 and CLIC5 leads to hearing loss in mice. **(A)** Representative ABR traces to click stimuli in wild-type (black traces), *Grxcr2*^–/–^ (green traces), and *Grxcr2^*D*180/D180^* mice (red traces). Note, *Grxcr2*^–/–^ mice had profound hearing loss while *Grxcr2^*D*180/D180^* mice have moderate hearing loss. **(B)** ABR thresholds for click stimuli in 6-week-old wild-type, *Grxcr2*^–/–^ and *Grxcr2^*D*180/D180^* mice. All values are represented as the mean ± SEM. ***p < 0.001, Student’s *t* test. **(C)** ABR thresholds for pure tones in 6-week-old wild-type, *Grxcr2*^–/–^ and *Grxcr2^*D*180/D180^* mice. *P* < 0.001 between wild-type and *Grxcr2*^–/–^ mice, wild-type and *Grxcr2^*D*180/D180^* mice, *Grxcr2*^–/–^ and *Grxcr2^*D*180/D180^* mice (two-way ANOVA). **(D)** Model of GRXCR2 protein complex at the base of stereocilia. In our model, GRXCR2 forms a protein complex with CLIC5 and taperin, which is surrounded by a circumferential ring structure formed by RIPOR2 (previously named as Fam65b) oligomers. GRXCR2 and CLIC5 regulate the localization of taperin, which is an actin cytoskeleton regulatory protein located at the base of stereocilia.

To investigate whether the interaction between GRXCR2 and CLIC5 is required for auditory perception, brain stem response (ABR) to broadband click stimuli in 6-week-old animals was measured. The *Grxcr2*^–/–^ mice had profound hearing loss. Interestingly, although the *Grxcr2^*D*180/D180^* has fairly normal V-shaped hair bundles, they had a moderate hearing loss with ∼20 dB hearing threshold elevation compared with wild-type mice (Figures 4A,B). Measurements of responses to pure tones revealed that *Grxcr2^*D*180/D180^* had moderate hearing loss at lower frequencies (∼10–15 dB hearing thresholds elevation at 8 and 12 kHz) and more severe hearing loss at higher frequencies (∼40 dB hearing thresholds elevation at 24 and 28 kHz) (Figure 4C). These results suggest that the interaction between GRXCR2 and CLIC5 is required for normal hearing especially for hearing at high frequencies (Figure 4D).

## Discussion

The basal region of stereocilia is critical for hair cell function and many deafness-related proteins are concentrated at this region. Here, we revealed an interaction between GRXCR2 and CLIC5, two proteins linked to hearing loss in humans (Imtiaz et al., 2014; Seco et al., 2015; Wonkam et al., 2021). In our previous studies, we found that GRXCR2 forms a complex with taperin at the base of stereocilia. Depleting GRXCR2 expression leads to profound hearing loss which is partially caused by the mislocalization of taperin in the hair cells, as reducing taperin expression level only partially restores the hearing in null *Grxcr2*-mutant mice (Liu et al., 2018). Hearing thresholds are still significantly elevated especially at high frequencies after the morphological defects of stereocilia are rescued in the *Grxcr2*^–/–^ mice, suggesting that GRXCR2 interacts with additional proteins required for normal hearing (Liu et al., 2018). Indeed, here we revealed an interaction between GRXCR2 and CLIC5, which is also essential for normal hearing.

Glutaredoxin domain-containing cysteine-rich protein 2, CLIC5, and taperin form a protein complex at the base of stereocilia (Salles et al., 2014; Bird et al., 2016, 2017; Liu et al., 2018). By mapping their binding domains, we found that different regions of GRXCR2 mediate the interaction with CLIC5 and taperin, respectively. Although the interaction between GRXCR2 with CLIC5 is not critical for the stereocilia morphogenesis, loss of this interaction might affect the stability of the protein complex formed by GRXCR2, CLIC5, and taperin, and results in moderate hearing loss.

Chloride intracellular channel protein 5 has been linked to hearing loss in humans and mice but its functions in hair cells are still unknown (Gagnon et al., 2006; Seco et al., 2015). Structure analysis found that CLIC5 belongs to the glutathione S-transferase (GST) fold superfamily, however, evidences are still lacking to demonstrate a putative enzymatic function (Littler et al., 2010). Although some CLIC proteins could form integral membrane ion channels *in vitro* (Littler et al., 2010), studies have found that CLIC5 is tightly associated with the cytoskeleton, rather than functioning as an ion channel in hair cells (Salles et al., 2014). In line with that, taperin, an actin cytoskeleton regulatory protein in stereocilia (Rehman et al., 2010; Liu et al., 2018), directly binds to CLIC5 (Salles et al., 2014; Bird et al., 2017). Here, we revealed a novel interaction between CLIC5 and GRXCR2. Similar to the *Grxcr2*^–/–^ mice, *Clic5*-deficient mice have disorganized stereocilia, in which taperin is also diffused along the stereocilia (Salles et al., 2014). Since the mislocalized taperin causes the morphological defects of stereocilia in the *Grxcr2*^–/–^ mice (Liu et al., 2018), the morphological defects of stereocilia and hearing loss in *Clic5*-deficient mice might be also caused by or partially caused by the mislocalization of taperin. To test this hypothesis, further studies to investigate whether reducing taperin expression level could rescue or partially rescue the stereociliary morphological defects and/or hearing loss in *Clic5*-deficient mouse would be informative.

Glutaredoxin domain-containing cysteine-rich protein 2 is concentrated at the base of stereocilia. Although it interacts with taperin and CLIC5, depleting taperin or CLIC5 alone in hair cells does not affect the localization of GRXCR2 in hair cells. One possibility is that depleting taperin or CLIC5 alone is not enough to affect the localization of GRXCR2 in hair cells. We are currently crossing *Clic5* and *taperin* null mice and will investigate the localization of GRXCR2 in the *Clic5* and *taperin* double knockout mice. Another possibility is that some other protein(s) at the base of stereocilia determines the localization of GRXCR2. It will be of interest to screen additional GRXCR2 interacting proteins and investigate the extent to which they are required for GRXCR2 localization in hair cells.

## Data Availability Statement

The original contributions presented in the study are included in the article/Supplementary Material, further inquiries can be directed to the corresponding author/s.

## Ethics Statement

The animal study was reviewed and approved by Institutional Animal Care and Use Committee of The Scripps Research Institute and Indiana University School of Medicine.

## Author Contributions

All authors contributed to the methodology and investigation. JL and BZ: writing. BZ: conceptualization and supervision.

## Conflict of Interest

The authors declare that the research was conducted in the absence of any commercial or financial relationships that could be construed as a potential conflict of interest.
