# Supplementary material for: N-Terminus of GRXCR2 Interacts With CLIC5 and Is Essential for Auditory Perception
Source: Front Cell Dev Biol. 2021 May 5;9:671364. doi: 10.3389/fcell.2021.671364 (PMC8131845; doi:10.3389/fcell.2021.671364)
Supplement: Supplementary file 1 [file Data_Sheet_1.docx]

**
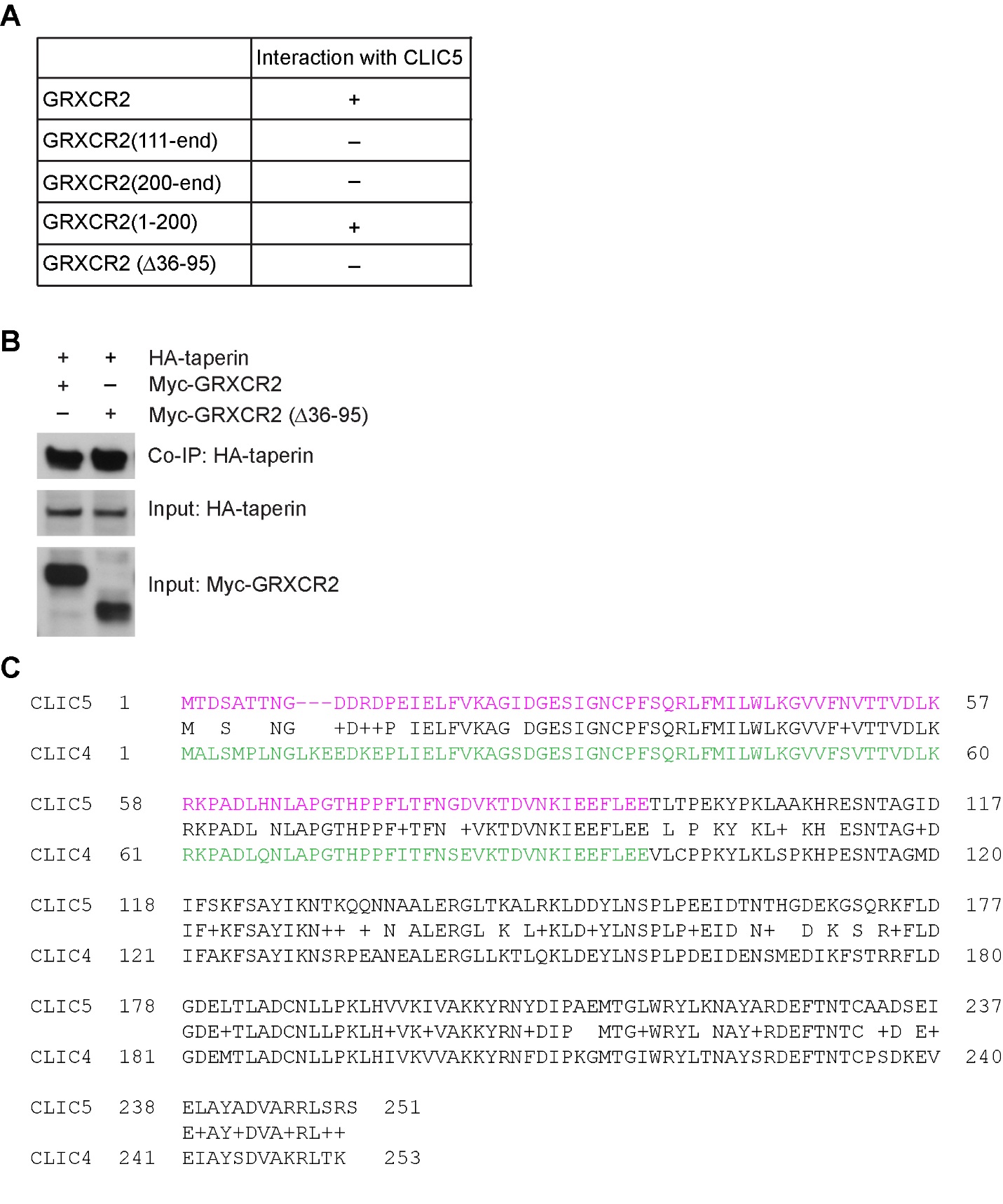
**

**Supplementary Figure 1: (A)** GRXCR2 constructs used for biochemical experiments. **(B)** GRXCR2 and GRXCR2(Δ36-95) interact with taperin. HEK293 cells were transfected with the constructs indicated on the top of each panel. Immunoprecipitations were carried out with Myc-antibody followed by western blotting to detect coexpressed proteins. The upper row shows Co-IP result and lower rows show input proteins. **(C)** Sequence alignment between CLIC5 and CLIC4. Note, the first 94 amino acids of CLIC5 and the first 97 amino acids of CLIC4 are highlighted in magenta and green.
